# Body packing by rectal insertion of cocaine packets: a case report

**DOI:** 10.1186/1756-0500-6-178

**Published:** 2013-05-03

**Authors:** Fabio Fernandes Neves, Palmira Cupo, Valdair Francisco Muglia, Jorge Elias Junior, Marcello Henrique Nogueira-Barbosa, Antonio Pazin-Filho

**Affiliations:** 1 Department of Medicine, Federal University of Sao Carlos, Sao Carlos, Brazil; 2 Department of Pediatrics and Puericulture, Faculty of Medicine of Ribeirao Preto, University of Sao Paulo, Ribeirao Preto, Brazil; 3 Department of Internal Medicine, Faculty of Medicine of Ribeirao Preto, University of Sao Paulo, Ribeirao Preto, Brazil

## Abstract

**Background:**

Body packing is used for international drug transport, immediate drug concealment during a police searching or introducing drugs inside prisons. Despite the high level of specialization of dealers who have started to manufacture more complex packs, up to 5% of patients could develop intoxication due to pack rupture. Bowel obstruction is another acute complication.

**Case presentation:**

A 27-year-old black male patient was sent to the hospital by court order for clinical evaluation and toxicological examination. The patient was conscious, oriented, had good color, normal arterial pressure and heart rate, and no signs of acute intoxication. Abdominal examination revealed discrete pain upon deep palpation and a small mass in the left iliac fossa. A plain abdominal radiograph revealed several oval structures located in the rectum and sigmoid. Fasting and a 50 g dose of activated charcoal every six hours were prescribed. After three days, the patient spontaneously evacuated 28 cocaine packs.

**Conclusion:**

Adequate clinical management and prompt identification of potential complications are of fundamental importance in dealing with body packing.

## Background

Body packing is a way to deliver drugs across international borders in the form of packages hidden in anatomical cavities such as the mouth, rectum, intestine, ear and vagina [1,2].

In addition to the use in international transport, this method is also used to introduce illicit drugs into prisons and in the compulsive attempt to conceal a situation of flagrante delicto during a police search, also called body stuffing [2].

As much as one kg of illicit drugs divided into large numbers of packs can be hidden during transportation. The drugs most commonly transported are cocaine, heroin, and cannabis derivatives [1]. Each heroin or cocaine pack exceeds the lethal dose by several times for human beings, with reported cases of sudden death having been reported in the occurrence of pack rupture [3]. Mechanical complications such as obstruction, bleeding and intestinal perforation have also been reported [3,4].

Cases reported in the literature originate from large metropolitan regions or from areas close to customs facilities. The present report documents a situation experienced in an emergency service in the interior of the state of São Paulo, Brazil, a fact that may indicate geographical expansion of this type of crime. The pertinent clinical, diagnostic, therapeutic and ethical aspects of the case are discussed.

## Case presentation

A 27-year-old black male patient was detained by the Federal Police for suspected drug trafficking. He was believed to have introduced cocaine packs into his rectum. He was sent to the hospital by court order for clinical examination and toxicological evaluation.

The patient was conscious, oriented, had good color, normal blood pressure and heart rate, and no signs of acute intoxication. Abdominal examination revealed discrete pain upon deep palpation and a small mass in the left iliac fossa, although with no signs of an acute abdomen. The patient didn’t consent to rectal examination.

**Figure 1:**
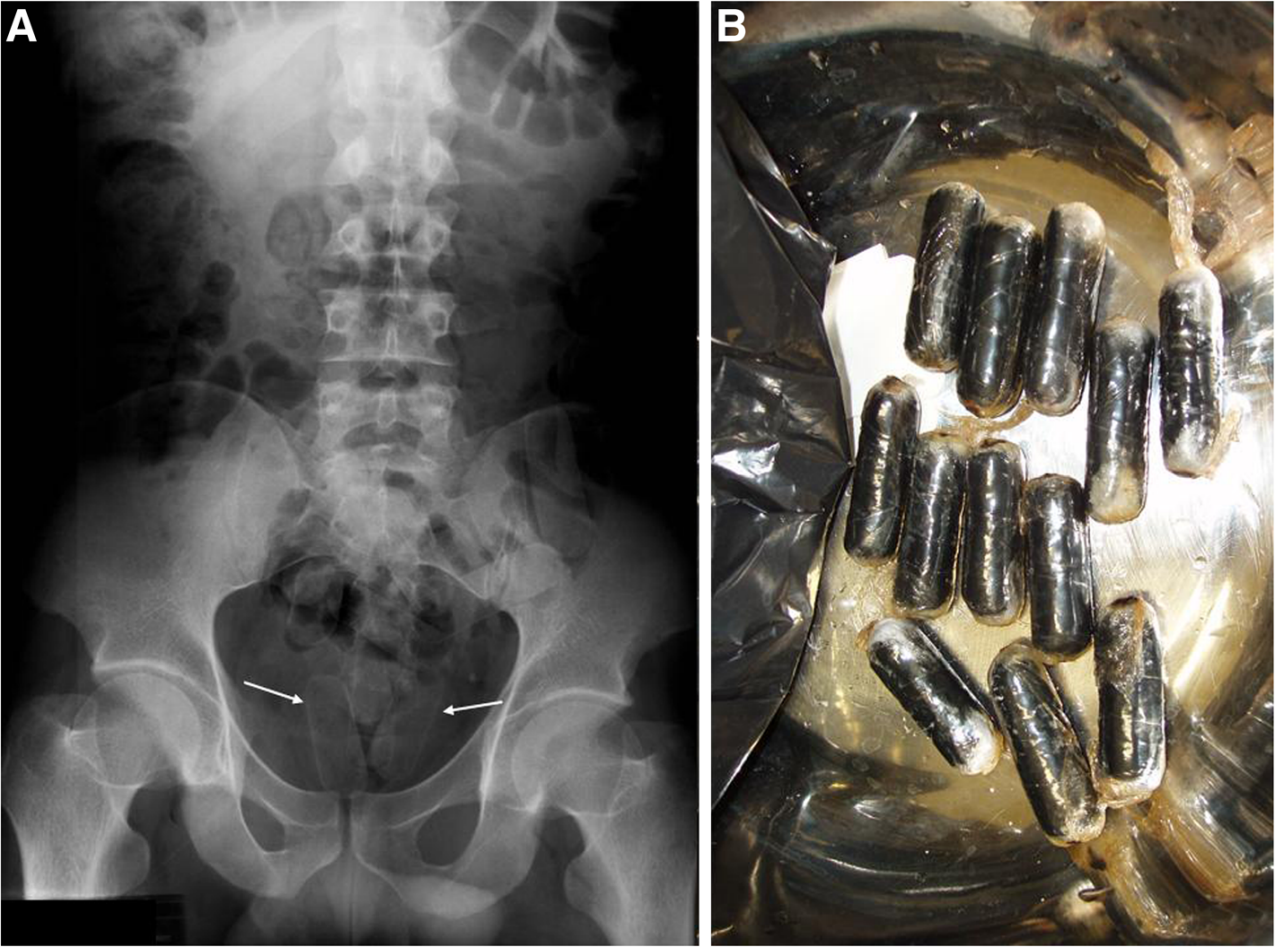
**Cocaine packages hidden in the patient intestine. A**- Plain abdominal radiograph performed on the day of patient admission showing several foreign bodies located in the sigmoid colon (white arrows); **B**- Small cocaine packages evacuated by the patient.

Determination of serum benzoylecgonine (a cocaine metabolite) was negative. A plain abdominal radiograph revealed several oval structures located in the rectum and sigmoid colon (Figure 1).

Fasting and whole bowel irrigation with an osmotically balanced polyethylene glycol electrolyte solution and a 50 g dose of activated charcoal every six hours were prescribed and the patient was kept under hospital observation. During the next three days, the patient spontaneously evacuated 28 cocaine packs which were sent to the Police Department for criminal forensic examination (Figure 1). A control abdominal radiograph didn’t show any other foreign bodies. The forensic police identified around 30 g of cocaine in each package.

After the terrorist attacks of 2001, surveillance at border posts, airports and seaports were reinforced, with the adoption of image synthesizing devices for the check of passengers and baggage [1,3]. These measures have led traffickers to modify the process of drug pack preparation. What used to be prepared in a handmade manner started to be elaborated in a more sophisticated manner including the use of several layers of radiotransparent material such as latex, plastic, cellophane and carbon paper [1].

The method of imaging most accessible for the identification of drug packs is plain radiograph, which has a sensitivity of 85-90% [3]. In general, when drugs are swallowed, multiple foreign bodies of a spherical shape and with a diameter of less than 2 cm are observed in the small bowel. In contrast, when the drug is inserted into the rectum, a smaller number of oval packs reaching as much as 6 cm in length are identified [1]. It is clear that the sensitivity of the radiologic method can be influenced by the number of packs ingested or introduced and by the radiologic density of the drug.

Hashish has a denser attenuation coefficient than feces and heroin and a density similar to that of air, which permits a relatively easy identification of this drug. However, the radiological density of cocaine is closely similar to that of feces; a fact that impairs radiological analysis [1]. In sporadic and specific situations, computed tomography can be used to obtain greater information about ingested content, such as when is suspected that the number of packages may have been underestimated by the patient.

In general, asymptomatic patients are taken to the hospital by court or police order. These patients must be continuously monitored since spontaneous pack rupture with acute intoxication occurs in up to 5% of cases [3]. Intestinal irrigation with polyethylene glycol can be prescribed in order to reduce the risk of rupture [1]. Monitoring time is extremely variable since many patients ingest substances that reduce intestinal transit in order to prevent evacuation during transportation of the drug. Any type of prokinetic medication for the induction of evacuation should be avoided during the period of observation since these medications may facilitate rupture of the drug packs in the presence of the mechanical effort of intestinal contraction [3].

Clinical manifestations, when present, may be due to gastrointestinal complications or to systemic intoxication.

Intestinal obstruction is the most frequent surgical complication in drug smuggling patients. In a series of 70 operations performed on body packers in the Caribbean, signs of peritoneal irritation were observed in 57 patients and uncontrollable vomiting in 45 [4].

Heroin and cocaine are the drugs most frequently detected in cases of acute smuggler intoxication. Patients intoxicated with heroin present with reduced consciousness, bradypnea and pupillary miosis. They should receive ventilator and circulatory support, as well as 2 to 5 mg naloxone every 5 minutes until reversal of symptoms. High doses of this antidote may be necessary in the case of rupture of intestinal packs [5].

The most common clinical presentation of acute cocaine intoxication is adrenergic hyperactivation characterized by tachycardia, arterial hypertension, mydriasis, and psychomotor agitation. Cardiovascular complications such as myocardial infarction, tachydysrhythmia and aorta dissection are leading causes of death among victims of a cocaine overdose. Since there is no effective cocaine antidote, when rupture of an intestinal pack is suspected the patient should be immediately submitted to laparotomy for removal of that packets [3]. Palliative treatments such as benzodiazepines for the control of seizures or psychomotor agitation, lidocaine for ventricular dysrhythmias and sodium nitroprusside for the control of severe arterial hypertension can be prescribed [6].

In this situation, the patient may refuse physical examination such as digital rectal examination or complementary tests that might incriminate him. From an ethical viewpoint, it is the responsibility of the doctor to inform the patient about the risks of his physical situation while respecting his decision. The patient should be kept in the hospital under intensive monitoring of signs of acute intoxication.

## Conclusion

In view of the wide geographic dissemination of drug traffic and of the use of the human body for the transport of these substances, emergency physicians should keep in mind this differential diagnosis in cases of circulatory collapse or consciousness of no apparent cause.

## Consent

Written informed consent was obtained from the patient for publication of this case report and accompanying images. A copy of the written consent is available for review by the Editor-in-Chief of this journal.

## Competing interests

The authors declare that they have no competing interests.

## Authors’ contributions

VFM, JEJ and MNB analyzed and interpreted the patient data regarding the radiological procedures. PC gave toxicological support during patient care. FFN and APF gave clinical support during patient care. All authors contributed to the writing of the manuscript and approved the final version.
